# Are serum cystatin-C-based estimates better than those derived from serum creatinine in critically ill patients?

**DOI:** 10.1186/cc10963

**Published:** 2012-03-20

**Authors:** JP Baptista, SC Teixeira, J Pimentel

**Affiliations:** 1 Coimbra Universitary Hospital, Coimbra, Portugal

## Introduction

The aim of our study was to evaluate the utility of two cystatin-C-based equations, as a surrogate of the renal function (glomerular filtration rate (GFR)) in a group of critically ill patients.

## Methods

This was a monocentric, prospective and observational study including 146 samples respecting 22 ICU patients. Daily evaluation of seric creatinine, seric cystatin C (CC) and 24-hour creatinine clearance (24CrCL) was performed during the ICU stay. Comparisons were done between two CC-based equations (Hoek (H) and Larsson (L) formulas) and: 24CrCL; Cockroft-Gault (CG); modified Cockroft-Gault (mCG); and six-variable Modification of Renal Disease (MDRD6) formulas. Patients with chronic renal failure were excluded. Correlation, precision, bias and discrimination power were assessed using Spearman coefficient, Bland-Altman plots and receiver operating characteristic curves.

## Results

The average age of the patients was 63.4 years and male gender was predominant (68%). The APACHE II score was 16.8 ± 5.7. The medians of H and L estimates were 50.5 (28 to 77.6) and 47.7 (24.5 to 79.2) respectively, as compared to 69.8 (29.8 to 115.7), 60.7 (42.6 to 101.4), 58.9 (42.6 to 65.1) and 59.2 (40.6 to 106.8) ml/minute/1.73 m^2^, respectively to 24CrCL (reference method), CG, mCG and MDRD6. Correlation (*r*) between H, F, CG, mCG, MDRD6 and 24CrCL was 0.83/0.83/0.73/0.70/0.74, respectively. H and L formulas showed the smallest bias and limits of agreement, when compared with formulas based on serum creatinine, respectively -17.5/±52 ml/minute/1.73 m^2 ^and -21/±52.8 ml/minute/1.73 m^2^. The sensibility for the identification of acute renal dysfunction (24CrCL under 60 ml/minute/1.73 m^2^) was high for H and L formulas (area under the curve of 0.94 for both). In the subgroup of 29 samples with 24CrCL above 130 ml/minute/1.73 m^2 ^(patients with hyperfiltration) these two formulas had low sensibility (between 8 and 22%) for identification of this condition.

## Conclusion

In this population of critically ill patients, cystatin-C-derived Hoek and Larsson equations underestimated 24CrCL; however, they have a better performance than the classic estimates (CG and MDRD6). Nevertheless, they are inaccurate when applied to ICU patients with hyperfiltration (GFR >130 ml/m/1.73 m^2^).

